# Geriatric cardiology in one’s own backyard?

**DOI:** 10.1007/s12471-023-01841-9

**Published:** 2023-12-19

**Authors:** Martin E. W. Hemels, Gerard J. Blauw

**Affiliations:** 1 Department of Cardiology, Rijnstate Hospital Arnhem, Arnhem, The Netherlands; 2 Department of Cardiology, Radboud University Medical Center Nijmegen, Nijmegen, The Netherlands; 3 Department of Internal Medicine-Geriatrics, Haaglanden Medical Center, Den Haag, The Netherlands; 4 Department of Internal Medicine-Geriatrics, Leiden University Medical Center, Leiden, The Netherlands

Both demographic and societal changes are responsible for the increasing number of older people admitted to hospitals, often in an acute setting [1]. A large proportion of this group, up to 40%, is admitted with cardiac problems. These patients are often frail with multiple conditions and diseases causing an excessive burden on available resources [1–4]. This would not be a major issue if the outcome of these emergency admissions would be favourable for the patient in question. Sadly, this is often not the case. It has been shown that the outcome is often detrimental, with figures up to 50% mortality and 70% functional decline after the first year of admittance [5]. Main causes for functional decline during hospitalisation are delirium and loss of muscle mass (sarcopenia), increasing length of stay (LOS) and more need for rehabilitation care after discharge. Even though the topic is extremely important, with an ageing population, literature on the subject is scarce.

In this issue, Raijmann et al. introduce geriatric co-management as a proof of concept to reduce complications and LOS of vulnerable older patient admitted at cardiology wards [6]. The results suggest a favourable effect of geriatric co-management, resulting in a 20% reduction in LOS and 50% more patients discharged to geriatric rehabilitation centres compared with historic controls (2016–2018). However, since COVID-19 changed the world dramatically, this also is the main weakness of the study, and therefore the favourable results found in the intervention group (2018–2020) may not fully reflect the current situation. Nevertheless, these figures cannot be ignored and require at least a new study with a proper control group in this post-COVID era to confirm the findings.

It’s beyond discussion that a reduction in LOS is favourable for both patients and the health-care system. In the present study, a mean reduction of one day (20%) was achieved in the group with geriatric co-management. This is a significant effect, but whether it is clinically relevant for the individual patient is the crucial question. Although intuitively the answer to this question is yes, complications during the admission e.g., the incidence of delirium and functional decline, were not investigated. Since this geriatric co-management costs extra manpower, which cannot be used for other hospitalised frail patients, these clinical endpoints should be addressed in future studies.

The finding that the reduction in LOS was accompanied by an increase in the number of patients discharged to geriatric rehabilitation centres is without any doubt due to the geriatric intervention, since geriatricians are familiar with the indications for this type of multidisciplinary rehabilitation. The finding that, compared with the controls, less patients were discharged to their own homes, suggests that transition to geriatric rehabilitation centres is underlying the observed reduction in LOS. Although again intuitively a rehabilitation period should be favourable, data for geriatric rehabilitation in this cardiovascular patient group are not available [7]. The fact that no effect on readmission was found—despite the increased discharge to geriatric rehabilitation centres—shows the necessity to examine the effects of a geriatric rehabilitation cardio-programme on clinical endpoints, quality of life measures and socioeconomic endpoints. We need new research into integrated chain care, especially for the group of vulnerable old patients with multimorbidity, to evaluate this complex and costly type of care. The present study results provide an impetus for this type of integrated chain care research.

However, in order to avoid hospital admissions, the main focus for the future should be to identify vulnerable older patients in the community or in an outpatient setting so we can apply preventive measures at an early stage [1, 8–10]. For this purpose, the development of novel risk scores might also help to discriminate between specific patient populations (e.g. ongoing trial: the Dutch-GERAF study, NCT05337202). Recurrent attention to patients with polypharmacy, and the development, evaluation and implementation of advanced care planning and palliative care will also contribute. In addition, as Ronin Collins also recently wrote, working together in a multidisciplinary approach, including informal care, helps to ensure the realisation of ‘life to years’ [11]. Ultimately, more focus on informal care and family care is indispensable, among other things, leading to care situated in one’s own backyard [12].
